# Preservation of circulatory death donor Rat hearts using hemoglobin-based oxygen carriers for normothermic machine perfusion: enhancing viability and functionality

**DOI:** 10.3389/fcvm.2026.1712642

**Published:** 2026-02-23

**Authors:** Zekai Huang, Xuan Pan, Xiangmeng Wang, Jianqiang Ji, Chuangjie Niu, Liwei Xu, Jun Lu, Shaoyi Zheng, Pengyu Zhou, Zhong Zhang

**Affiliations:** 1 Department of Cardiovascular Surgery, Nanfang Hospital, Southern Medical University, Guangzhou, China; 2 Department of Human Anatomy, School of Basic Medical Sciences, Southern Medical University, Guangzhou, China; 3 Department of Cardiovascular Surgery, Yuebei People’s Hospital Affiliated to Shantou University Medical College, Shaoguan, China

**Keywords:** donation after circulatory death, *ex vivo* heart perfusion, hemoglobin-based oxygen carriers, ischemia- reperfusion injury, myocardial preservation

## Abstract

**Introduction:**

Ischemia-reperfusion injury (IRI) remains a critical barrier to successful transplantation of donation after circulatory death (DCD) hearts, compromising myocardial viability through oxidative stress, inflammation, and apoptotic pathways. Hemoglobin-based oxygen carriers (HBOCs) are being explored as substitutes for blood and red blood cells (RBCs) in *ex vivo* heart perfusion (EVHP) to mitigate myocardial IRI.

**Methods:**

This study compared the efficacy of HBOC vs. conventional blood or RBC perfusion in attenuating IRI using a rat DCD heart EVHP and transplantation model. Donor hearts were perfused *ex vivo* for four hours with blood, RBCs, or HBOC. Myocardial function was assessed by hemodynamic parameters, blood gas analysis, and biomarkers of oxidative stress, apoptosis, and inflammation. Histological and molecular analyses were performed after transplantation.

**Results:**

HBOC perfusion significantly reduced myocardial IRI and inflammation, with lower levels of 4-hydroxynonenal (4-HNE) and pro-inflammatory cytokines compared with blood perfusion. Hemodynamic performance, including developed pressure and ±dp/dt, was superior in the HBOC group. In contrast, blood-perfused hearts exhibited elevated potassium and lactate levels, indicating ongoing injury. Post-transplantation analyses demonstrated reduced structural damage and inflammatory infiltration in HBOC-treated hearts.

**Discussion:**

These findings indicate that HBOC *ex vivo* perfusion exerts dual cytoprotective effects by limiting ischemic injury through optimized oxygen delivery and attenuating reperfusion-associated injury cascades via antioxidant and anti-inflammatory mechanisms. EVHP with HBOC represents a promising preservation strategy for DCD hearts, with the potential to reduce myocardial IRI and improve post-transplant cardiac function in marginal donor hearts.

## Introduction

Donation after circulatory death (DCD) have emerged as a vital source to expand the donor pool amidst the global shortage of transplantable organs (1). However, DCD hearts face extra challenges due to the ischemic insult sustained during the agonal phase, which compromises organ viability and function (2–4). A major contributor to this injury is ischemia-reperfusion injury (IRI), a complex pathological process triggered when blood supply is restored to ischemic tissue. IRI leads to a burst of reactive oxygen species (ROS), calcium overload, endothelial dysfunction, and activation of inflammatory cascades, ultimately exacerbating myocardial damage and impairing graft outcomes after transplantation (5, 6). in heart transplantation, IRI has been closely associated with primary graft dysfunction (PGD), a leading cause of early morbidity and mortality post-transplant (7, 8).

Traditional methods of static cold storage, while effective for short-term preservation, are inadequate for mitigating IRI in DCD hearts (4, 9). *Ex vivo* heart perfusion (EVHP) has been proposed as a transformative preservation strategy for donor hearts, offering physiological conditions that enable metabolic support, functional assessment, and therapeutic intervention (10). EVHP has demonstrated promising potential in reducing IRI and extending the preservation window, particularly for high-risk DCD hearts.

Hemoglobin-based oxygen carriers (HBOCs) represent an innovative adjunct in organ preservation strategies. As acellular oxygen carriers, HBOCs offer unique advantages, including efficient oxygen delivery and a reduced risk of immunogenicity compared to traditional blood-based perfusates (11). Preliminary studies have shown that HBOCs can maintain cellular metabolism and reduce oxidative stress in various organ preservation settings (12–14). However, the application of HBOCs in EVHP for DCD hearts remains underexplored.

This study investigates the feasibility and efficacy of using HBOCs in EVHP to preserve DCD rat hearts. By integrating HBOCs into the perfusate, we aim to enhance oxygen delivery, mitigate IRI, and improve the post-preservation viability and functionality of donor hearts, contributing to the development of novel strategies for heart transplantation.

## Materials and methods

This study involved 78 male Lewis rats (250–300 g; 10–12 weeks old) procured from Charles River Laboratories (Beijing, China). All animals were housed and handled in compliance with the Guide for the Care and Use of Laboratory Animals (National Institutes of Health Publication No. 85-23, revised 1996). The animal research protocol was reviewed and approved by the Ethical Committee of the Laboratory Animal Research Center at Southern Medical University Nanfang Hospital (Approval Number: NFYY-2022-0371). The rats were maintained in temperature-regulated rooms (22 ± 2 ℃) with a 12-hour light-dark cycle, provided with food and sterilized water, and allowed to acclimate for one week.

### Experimental groups

**Figure 1:**
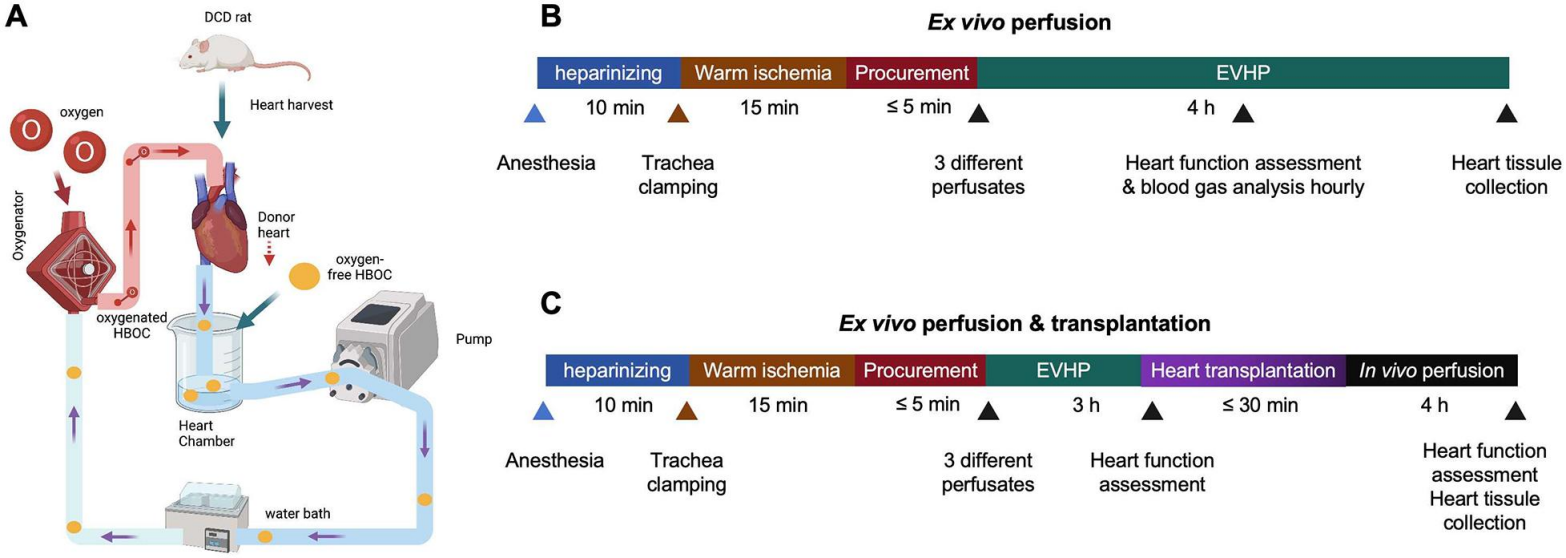
The schematic shows an overview of the protocol of the DCD model combined with normothermic EVHP and experimental design. **(A)** Protocol of the DCD model combined with normothermic EVHP. (**B** and **C**) Experimental design. DCD, donation after circulatory death; HBOC, hemoglobin-based oxygen carrier; EVHP, *ex vivo* heart perfusion.

The study was divided into two parts based on distinct experimental objectives. In the first part, DCD donor hearts underwent EVHP using three different perfusates. Perfusion was maintained for 4 h to assess sustained myocardial function recovery, metabolic stability, and perfusate blood gas parameters over time, during which left ventricular function and perfusate blood gas parameters were monitored hourly (Figure 1B). This extended perfusion period was designed to evaluate the ability of different perfusates to support prolonged normothermic preservation.

In the second part, DCD donor hearts were perfused using the same three perfusates for 3 h and subsequently transplanted into the abdominal cavity of recipient rats (Figure 1C). In this transplantation arm, EVHP served as a resuscitation and assessment phase prior to implantation, and a shorter perfusion duration was selected to balance adequate myocardial recovery with avoidance of excessive *ex vivo* exposure before transplantation. Each experimental group consisted of six animals (*n* = 6).

An appropriate amount of oxygen carrier was added to the modified Krebs-Henseleit (KH) solution in each group to achieve a perfusate hemoglobin concentration of 6 g/L. Hydroxyethyl starch (15 mg/mL) was added to the perfusate to maintain a colloid osmotic pressure of approximately 300 mmol/L.

To prepare blood-based perfusates, whole blood was collected from an additional syngeneic Lewis rat via the abdominal aorta into a heparinized syringe, yielding approximately 12–15 mL of whole blood per donor. The final perfusate volume for each EVHP experiment was 30 mL. For the Blood group, 6 mL of freshly collected whole blood was directly mixed with modified KH solution to reach the target hemoglobin concentration. For the RBC group, whole blood obtained from the same donor rat was centrifuge to separate plasma and cellular components; the RBC fraction was washed with 50 mL of normal saline and subsequently resuspended in modified KH solution to achieve the same target hemoglobin concentration of 6 g/L. This approach ensured that the Blood and RBC perfusates were derived from the same blood source and standardized by hemoglobin concentration.

In the HBOC group, polymerized bovine hemoglobin (HBOC; 5.4 g/dL; RedPharm, Beijing, China) was used as the oxygen carrier. Methylene blue (1.425 mg/mL) and glutathione (12.75 mg/mL) were added to the perfusate to reduce the formation of methemoglobin.

Accordingly, donor hearts were allocated into three groups based on the oxygen carrier used during perfusion: (1) HBOC group, in which HBOC served as the oxygen carrier; (2) Blood group, in which whole blood from the additional donor rat was used; and (3) RBC group, in which washed RBCs from the same donor rat were used as the oxygen carrier.

### DCD Heart procurement

The surgical procedure was adapted from previously published methods (15, 16). A 22G, 1-inch intravenous catheter was inserted into the right carotid artery and connected to a three-way stopcock to allow systemic heparin injection (2,000 IU/kg) and real-time perfusion pressure monitoring using a pressure transducer (AD Instruments Inc., USA). Although this heparin dose is higher than typically used in clinical practice, it was selected to ensure adequate anticoagulation in the small-animal circulation, minimize microthrombus formation, and prevent clotting during rapid organ procurement and subsequent connection to the EVHP circuit, where small-volume systems are particularly susceptible to clot-related flow instability.

Circulatory death was induced by clamping the trachea with mosquito forceps to initiate apnea. Death was confirmed when systolic pressure fell below 30 mmHg or when asystole occurred. The warm ischemic time (WIT) was defined as asystolic (post-circulatory-arrest) warm ischemic time and was initiated only after confirmation of circulatory arrest, rather than from the onset of tracheal clamping (functional WIT). A 15-minute asystolic WIT was applied in all donor hearts. Following the WIT period, a median sternotomy was performed, along with the ligation and dissection of the superior and inferior vena cava and the pulmonary veins. The aorta and pulmonary artery were isolated and severed, after which the aorta was connected to the *ex vivo* heart perfusion inlet. The heart retrieval process took no longer than 5 min.

### *Ex vivo* heart perfusion

Following 15 min of oxygenation for the perfusate, the donor heart was attached to the EVHP system through the aortic cannula (Figure 1A). The perfusion flow rate was established at 2.5 mL/min. Throughout the EVHP, the temperature of the isolated heart was maintained at 35–37 °C. The initial partial pressure of oxygen (PO_2_) ranged from 400 to 600 mmHg, while the pH was between 7.30 and 7.40.

### Cardiac functional assessment and blood gas analysis during EVHP

Following a 15-minute stabilization period, the latex balloon linked to a pressure sensor (ADInstruments Inc., USA) was introduced into the left ventricle via the left atrium. The assessment phase commenced at 1 h (T1) post-reperfusion and was evaluated hourly for a duration of 4 h (T2–T4). The cardiac functional parameters of the donor heart during EVHP comprised developed pressure (DP), heart rate (HR), dp/dt_max_ (maximum rate of rise of left ventricular pressure), and dp/dt_min_ (maximum rate of pressure decline). Additionally, blood gas analysis of the perfusate was conducted at each time point utilizing a blood gas analysis instrument (Gem Premier 3,500; Instrumentation Laboratory; USA).

### Heterotopic abdominal heart transplantation

The surgical procedure was referred to previously published articles (17, 18). In brief, after 3 h of reperfusion, the donor heart was removed and perfused with 4 °C cardioplegic solution (HTK, Fuzhou Neptunus Fuyao Pharmaceuticals Co. China) at a perfusion pressure of 60 cmH_2_O for 5 min. Following perfusion, the left atrial appendage was ligated using a 3-0 silk suture. The recipient rat was anesthetized with isoflurane gas, and a laparotomy was performed to expose and isolate the abdominal aorta and inferior vena cava. Using an 8-0 Prolene suture, the donor heart's aorta and pulmonary artery were anastomosed end-to-side to the recipient's abdominal aorta and inferior vena cava, respectively.

### Cardiac functional assessment after heart transplantation

4 h after transplantation, a rat pressure-volume catheter (ADInstruments Inc., USA) was employed to assess the cardiac function of the donor heart. The catheter was inserted into the left ventricle via the apex. The pressure was conveyed from the blood fluid to a pressure sensor, which converted the pressure signal into an electrical signal for the PowerLab system (ADInstruments Inc., USA). The LVDP, dp/dt_max_, dp/dt_min_, rate-pressure product (RPP), and tissue arterial unloading (Tau) were continuously recorded and analyzed using LabChart Pro (ADInstruments Inc., USA).

### Methemoglobin percentage test

The percentage of methemoglobin (Met-Hb %) of the total Hb content was measured using a commercial kit (Solarbio Science & Technology Co., Ltd., Beijing, China). The proportion of methemoglobin in the perfusate was measured using visible spectrophotometry. Briefly, perfusate samples were collected and diluted appropriately with phosphate-buffered saline (PBS). The absorbance of the samples was measured at wavelengths of 630 nm, 576 nm, and 560 nm using a spectrophotometer. The relative concentration of methemoglobin was calculated based on the absorbance ratios at these wavelengths, following established spectrophotometric equations for hemoglobin derivatives. All measurements were performed in triplicate to ensure accuracy and reproducibility.

### Hematoxylin-eosin staining

Cardiac tissues were collected and fixed in 4% paraformaldehyde for 24 h, followed by paraffin embedding. Tissue sections, 5 μm thick, were prepared using a microtome. Cardiac morphology was assessed through hematoxylin-eosin (HE) staining, and pathological changes were evaluated using a grading system based on established methods (17, 18). For each heart section, four nonoverlapping visual fields were randomly selected under a light microscope. Two blinded pathologists independently assessed the histopathological scores, with the average score across the four fields recorded for each sample.

### TUNEL staining

Myocardial apoptosis was evaluated using the Terminal deoxynucleotidyl transferase dUTP nick end labeling (TUNEL) test, as per the instructions established by the manufacturer (Roche Holding, Basel, Switzerland). Four non-overlapping visual areas were randomly chosen for inspection under a light microscope for each heart segment. The average number of apoptotic cells was determined for each specimen across the four fields. The apoptotic index was calculated by representing the number of TUNEL-positive cells as a proportion of the total cell count.

### Western blotting

The left ventricle was harvested and preserved in liquid nitrogen for subsequent western blot analysis. Cardiac tissue was homogenized and lysed using RIPA lysis buffer supplemented with a protease inhibitor cocktail. Protein concentrations were determined using a BCA protein assay kit. Proteins were separated via sodium dodecyl sulfate-polyacrylamide gel electrophoresis (SDS-PAGE) and transferred to polyvinylidene difluoride (PVDF) membranes (Millipore, Billerica, MA, USA). The blots were incubated overnight at 4 °C with primary antibodies against Bax (Abcam, ab32503, 1:1,000), Bcl-2 (Abcam, ab194583, 1:1,000), Caspase-3 (Abcam, ab184787, 1:1,000), Cleaved-Caspase-3 (Abcam, ab256469, 1:000), IL-1β (Abcam, ab254360, 1:1,000), IL-6 (Abcam, ab83053, 1:1,000), and TNF-α (Abcam, ab183218, 1:1,000). Following washes with Tris-buffered saline containing 0.1% Tween-20 (TBST; 10 mM Tris-base, 100 mM sodium chloride, pH 7.5), the membranes were incubated with secondary antibodies at room temperature for 1 h. Protein bands were quantified using the Image Lab software.

### Immunohistochemistry staining

Myocardial tissue slices were fixed in 4% paraformaldehyde, embedded in paraffin, and sectioned into 4 μm-thick slices. Immunoreactivity for 4-Hydroxynonenal (4-HNE, 1:1,000, Abcam, ab46545, USA) was assessed using a diaminobenzidine (DAB) reaction. Randomly selected fields from each slice were examined under a standard light microscope in a blinded manner. Image analysis was performed using Image-Pro Plus software (Media Cybernetics, USA). Four random, non-overlapping regions of each cardiac tissue section were analyzed, and the mean value was calculated for each specimen. HNE expression was quantified by measuring the integrated optical density (IOD).

### Statistical analysis

The mean ± SEM encompasses all data. Data statistical assessment was performed using GraphPad Prism 10.0.3 (GraphPad Software, Inc., La Jolla, CA). The Shapiro–Wilk normality test was used to assess the normal distribution before doing statistical analysis. A nonparametric Mann–Whitney test was used when the normality evaluation yielded a negative result. A two-way mixed repeated measures ANOVA (independent variable: various perfusates; dependent variable: perfusion time points) was used to evaluate changes in heart function between the two groups. Histopathological score differences were assessed using chi-square and Fisher's exact tests. A threshold of statistical significance was set at *P* < 0.05.

## Results

### Cardiac functional assessment during EVHP

**Figure 2:**
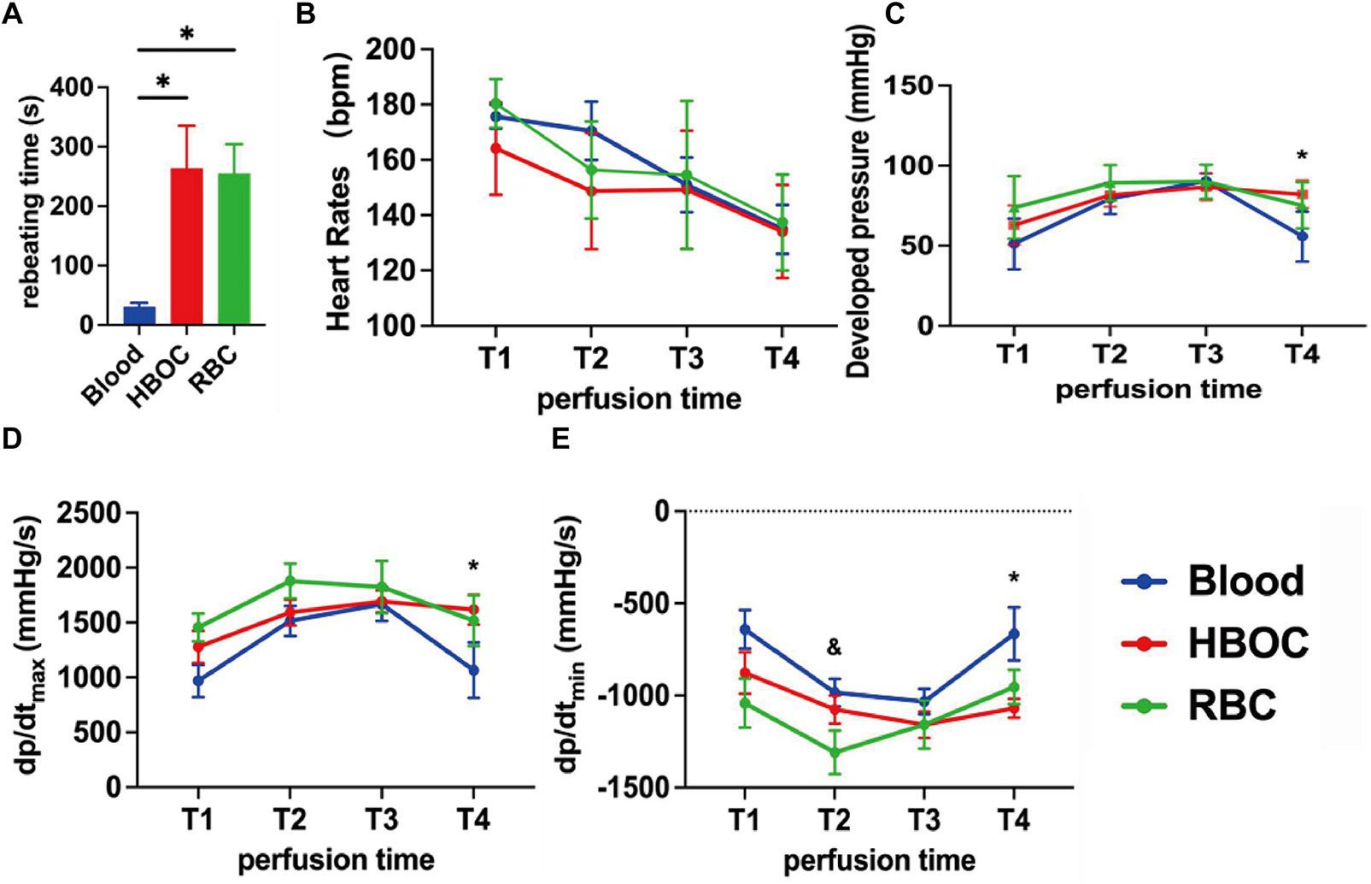
Cardiac functional assessment of the donor heart during EVHP. **(A)** donor heart rebeating time after reperfusion; **(B)** Heart Rate; **(C)** Developed pressure; **(D)** dp/dt_max_; **(E)** dp/dt_min_. HBOC, hemoglobin-based oxygen carrier; RBC, red blood cell; dp/dt_max_, maximum rate of rise of left ventricular pressure; dp/dt_min_, maximum rate of pressure decline. Data represent mean ± standard error of the mean (*n* = 6/group). *p < 0.05 blood vs. HBOC group, &*p* < 0.05 blood vs. RBC group.

Donor hearts exhibited distinct functional recovery patterns during *ex vivo* perfusion. Spontaneous contractions resumed rapidly in the blood group but were delayed in the HBOC and RBC groups (Figure 2A). HR gradually declined over time, with no significant intergroup differences at any time point (Figure 2B). Functional indices (DP, dp/dt_max_, and dp/dt_min_) improved progressively in all groups, peaking at 3 h before declining by 4 h. At 4 h, the blood group showed significantly lower DP, dp/dt_max_, and dp/dt_min_ than the HBOC group, while the RBC group showed no significant differences compared to either (Figures 2C–E).

### Perfusate gas analysis during EVHP

**Figure 3:**
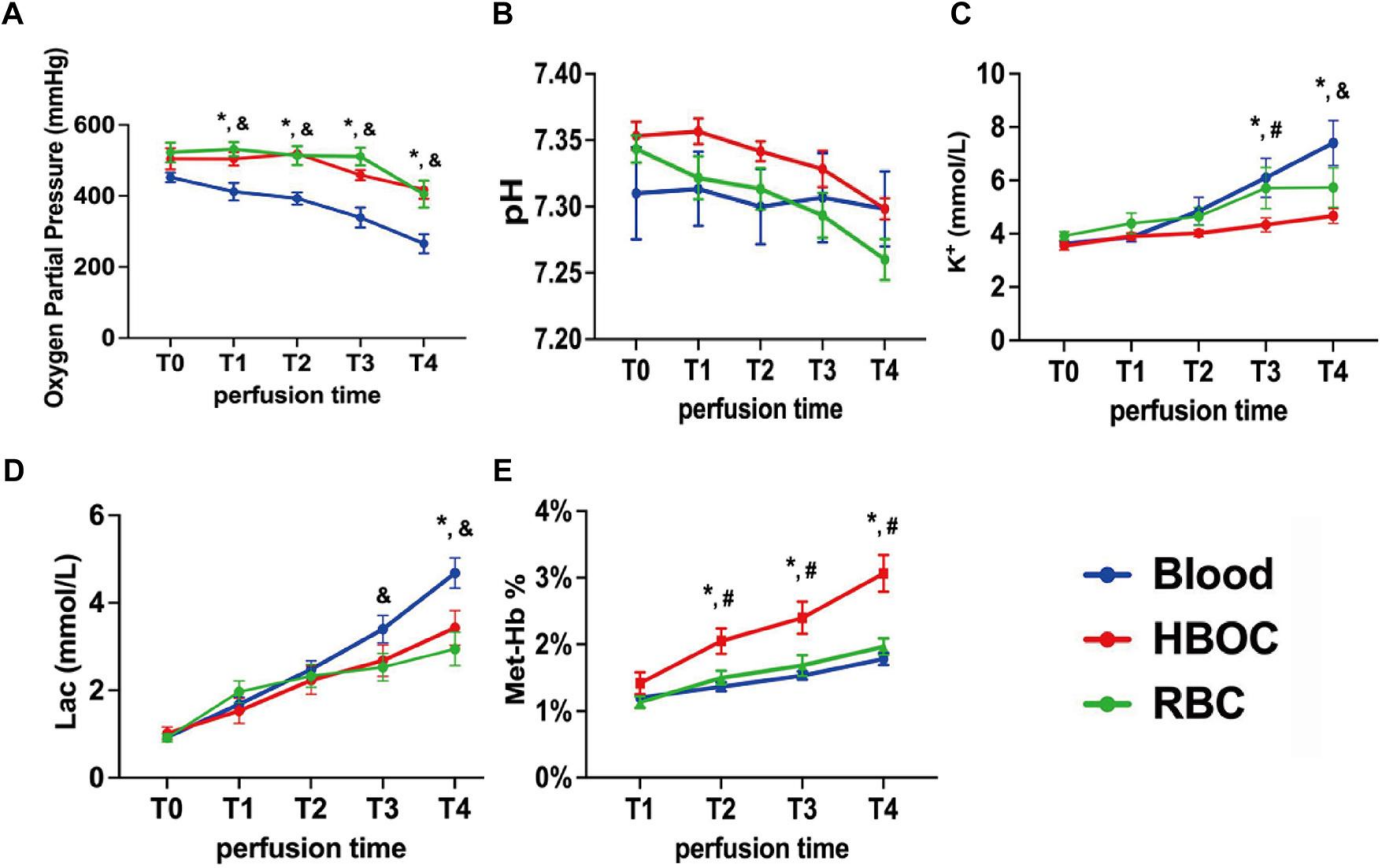
Perfusate gas analysis during EVHP. **(A)** Oxygen partial pressure; **(B)** pH; **(C)** K^+^ concentration; **(D)** Lactate concentration; **(E)** methemoglobin percentage. Lac, lactate; Met-Hb, methemoglobin. *n* = 6/group. *p < 0.05 blood vs. HBOC group, &*p* < 0.05 blood vs. RBC group, #*p* < 0.05 HBOC vs. RBC group.

Initial PO_2_ levels were comparable among groups but declined over time in the blood group, becoming significantly lower than in the HBOC and RBC groups (Figure 3A). The pH of the perfusate also declined over time, indicating progressive acidosis, with no significant intergroup differences (Figure 3B). K^+^ levels remained stable for 2 h but rose significantly in the blood and RBC groups from 3 h onward, with the blood group showing the highest level at 4 h (Figure 3C). Lactate increased over time, reflecting ongoing anaerobic metabolism, with significantly higher concentrations in the blood group at 3 and 4 h compared to the HBOC and RBC groups (Figure 3D).

Given HBOC's oxidation potential, Met-Hb levels were monitored. In the HBOC group, Met-Hb gradually increased but remained below 4% at 4 h, while blood and RBC groups consistently stayed at 1%–2% (Figure 3E).

### Inflammatory and oxidative stress markers after 4-hour perfusion

**Figure 4:**
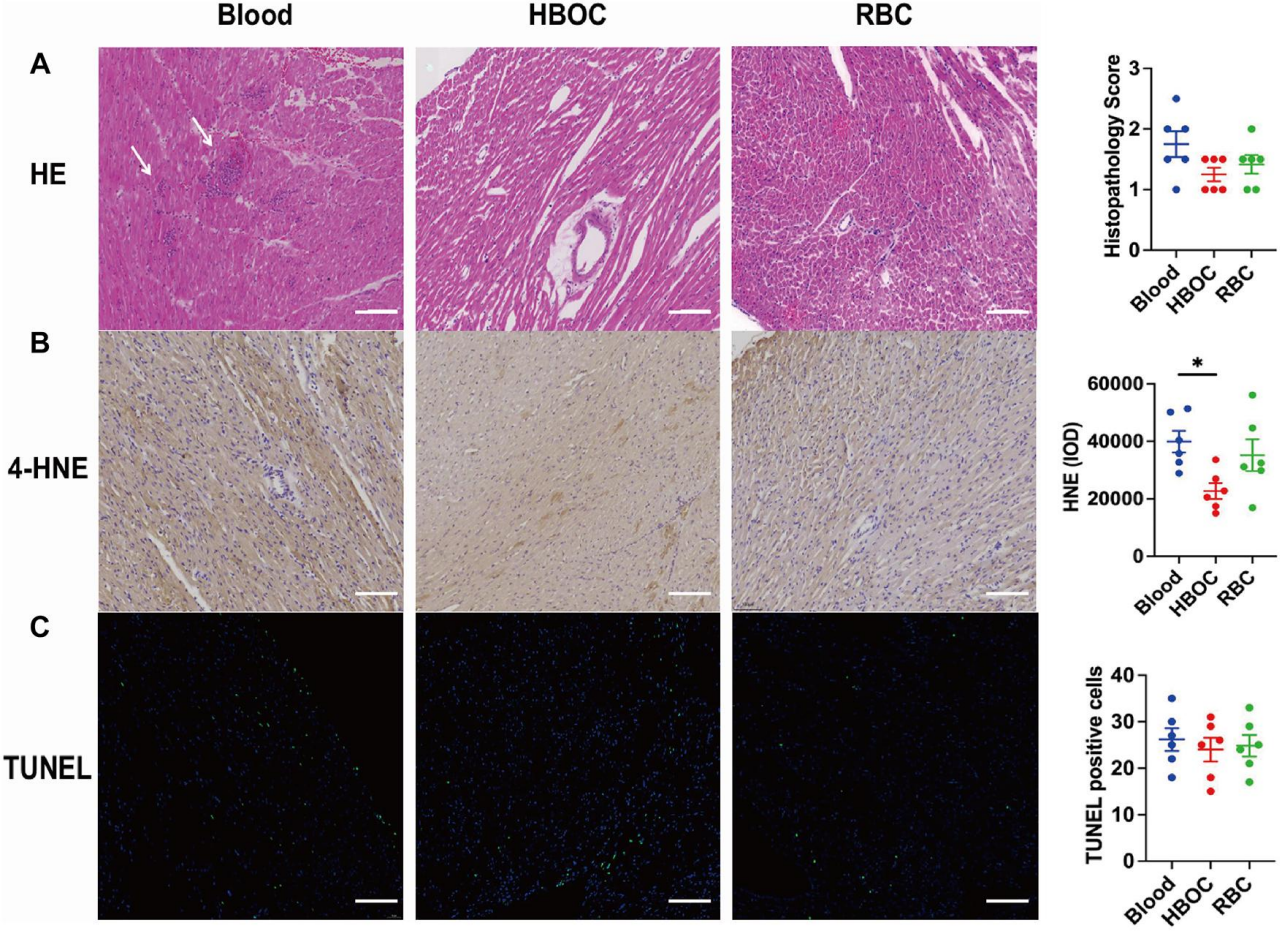
Histological evaluation post-EVHP **(A)** HE staining and histopathology score of myocardial tissue; **(B)** TUNEL staining with quantification; **(C)** 4-HNE immunohistopathology scores. scale ba*r* = 100 μm. HE, Hematoxylin-Eosin; TUNEL, Terminal deoxynucleotidyl transferase dUTP nick end labeling; 4-HNE, 4-Hydroxynonenal. *n* = 6/group. *p < 0.05.

**Figure 5:**
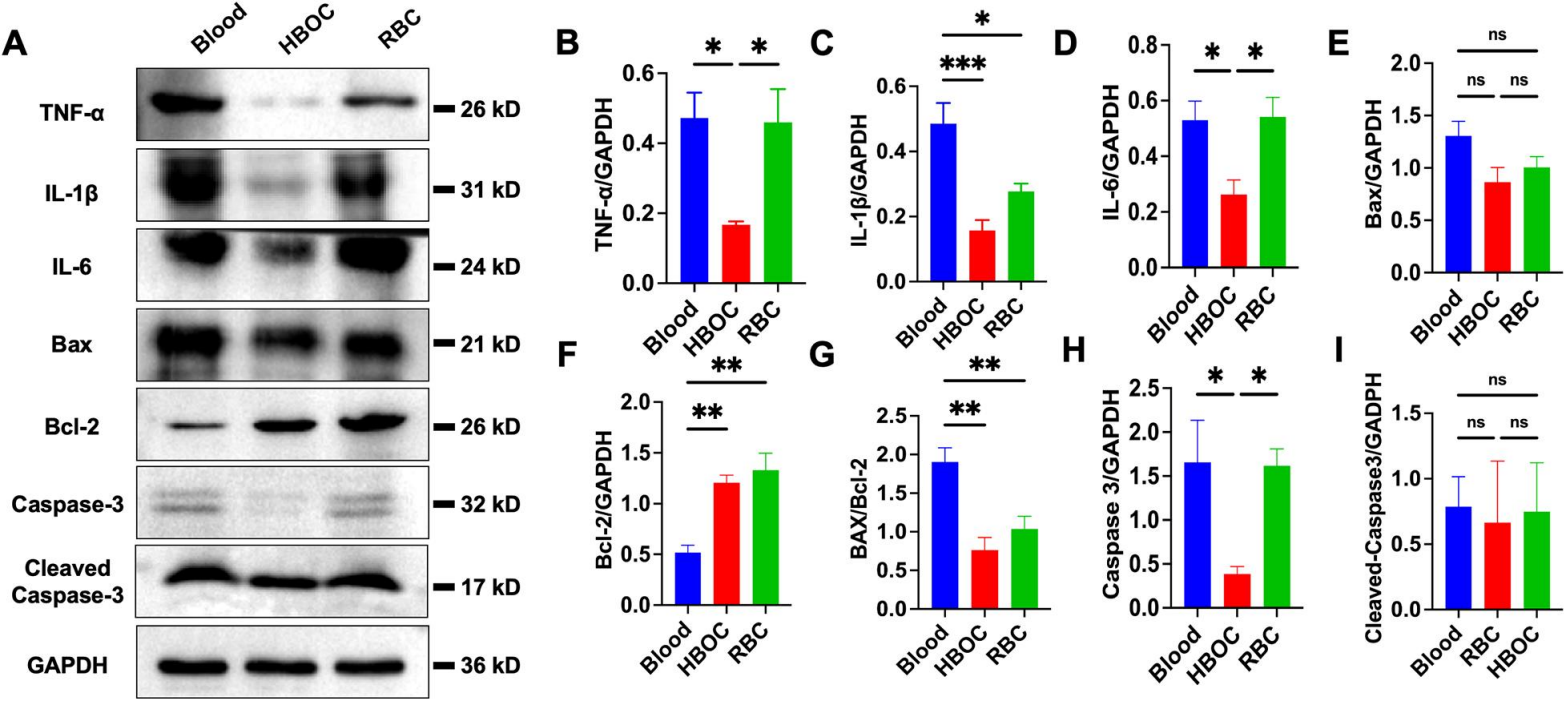
Western blot analysis post-EVHP. **(A)** Representative blots of TNF-α, IL-1β, IL-6, Bax, Bcl-2, caspase-3, and cleaved-caspase-3; **(B–I)** quantification of the protein expression. *n* = 6/group. *p < 0.05; **p < 0.01 for indicated comparisons.

After 4 h of perfusion, histology showed preserved myocardial fiber alignment and cellular integrity in all groups, though mild edema was present. The blood group exhibited marked inflammatory infiltration, as indicated by arrows in Figure 4A. Western blotting confirmed elevated TNF-α, IL-1β, and IL-6 levels in the blood group compared to HBOC and RBC groups (Figures 5A–D). Immunohistochemistry for 4-HNE indicated oxidative stress in all groups, with significantly higher 4-HNE expression in the blood group than in HBOC (Figure 4B).

### Assessment of myocardial apoptosis after 4-hour perfusion

TUNEL staining showed scattered apoptotic cardiomyocytes in all groups after 4 h of perfusion, with a slightly higher count in the blood group, though not statistically significant (Figure 4C). Western blotting confirmed expression of apoptosis-related proteins (Bax, Bcl-2, caspase-3, and cleaved-caspase-3) across groups (Figures 5A,E–I). The Bax/Bcl-2 ratio was lowest in the HBOC group (Figure 5H), while cleaved-caspase-3 levels were significantly elevated in the blood group vs. HBOC and RBC groups (Figure 5I).

### Post-transplant cardiac functional assessment

**Figure 6:**
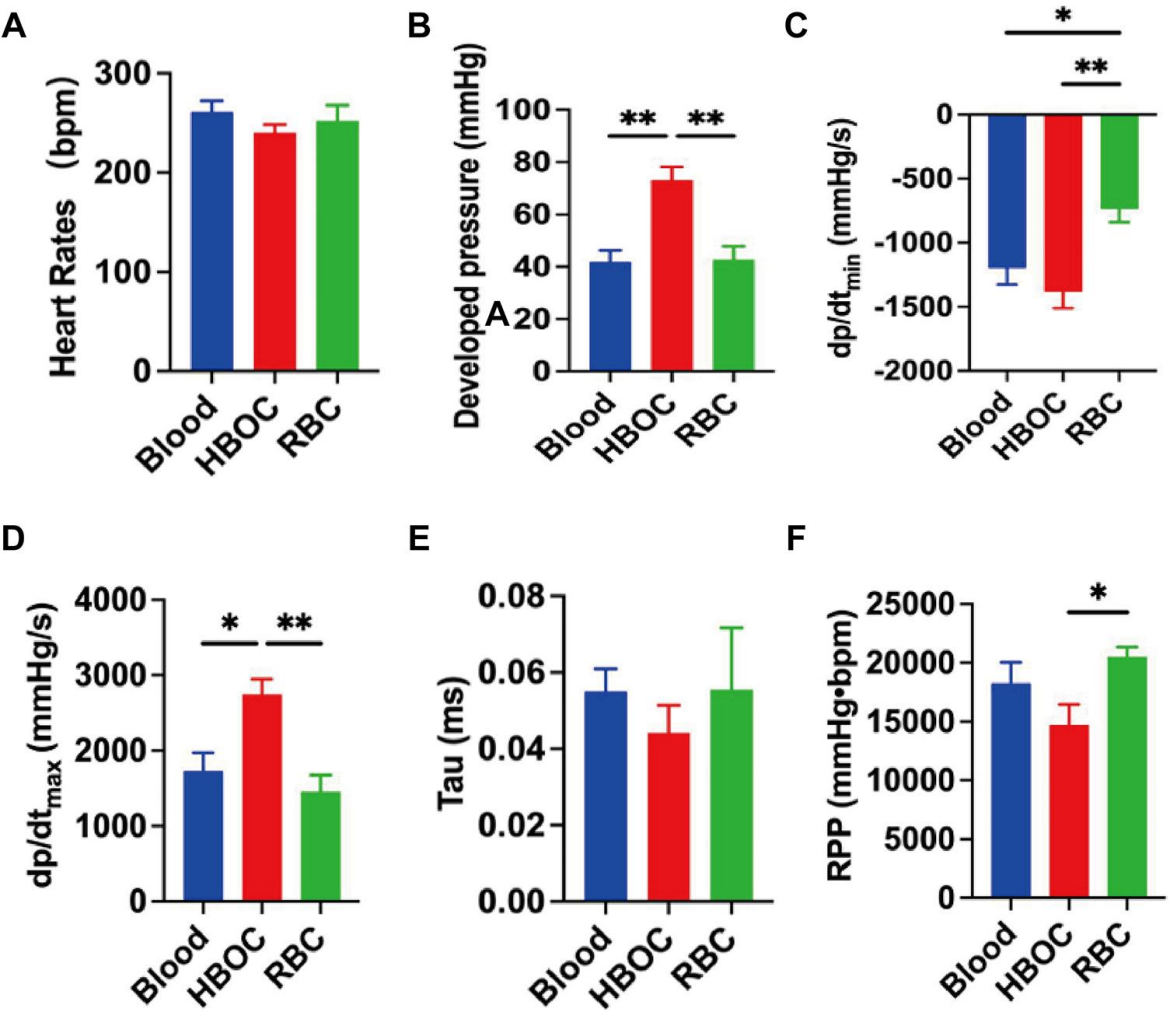
Cardiac functional post-transplantation. **(A)** Heart rates; **(B)** DP; **(C)** dp/dt_min_; **(D)** dp/dt_max_; **(E)** Tau; **(F)** RPP. Tau, tissue arterial unloading; RPP, rate pressure product. *n* = 6/group. *p < 0.05; **p < 0.01.

Cardiac functional evaluation was performed 4 h after donor heart transplantation. The results demonstrated no significant differences in heart rate among the three groups (Figure 6A). However, the HBOC group exhibited significantly superior performance in key cardiac function parameters, including DP, dp/dt_max_, and dp/dt_min_, compared to the blood and RBC groups (Figures 6B–D). No significant differences in Tau, an index of ventricular relaxation, were observed among the three groups (Figure 6E). Interestingly, the RPP, an indicator of myocardial workload, was higher in the RBC group compared to the HBOC group (Figure 6F).

### Histopathological and biochemical analysis post-transplantation

**Figure 7:**
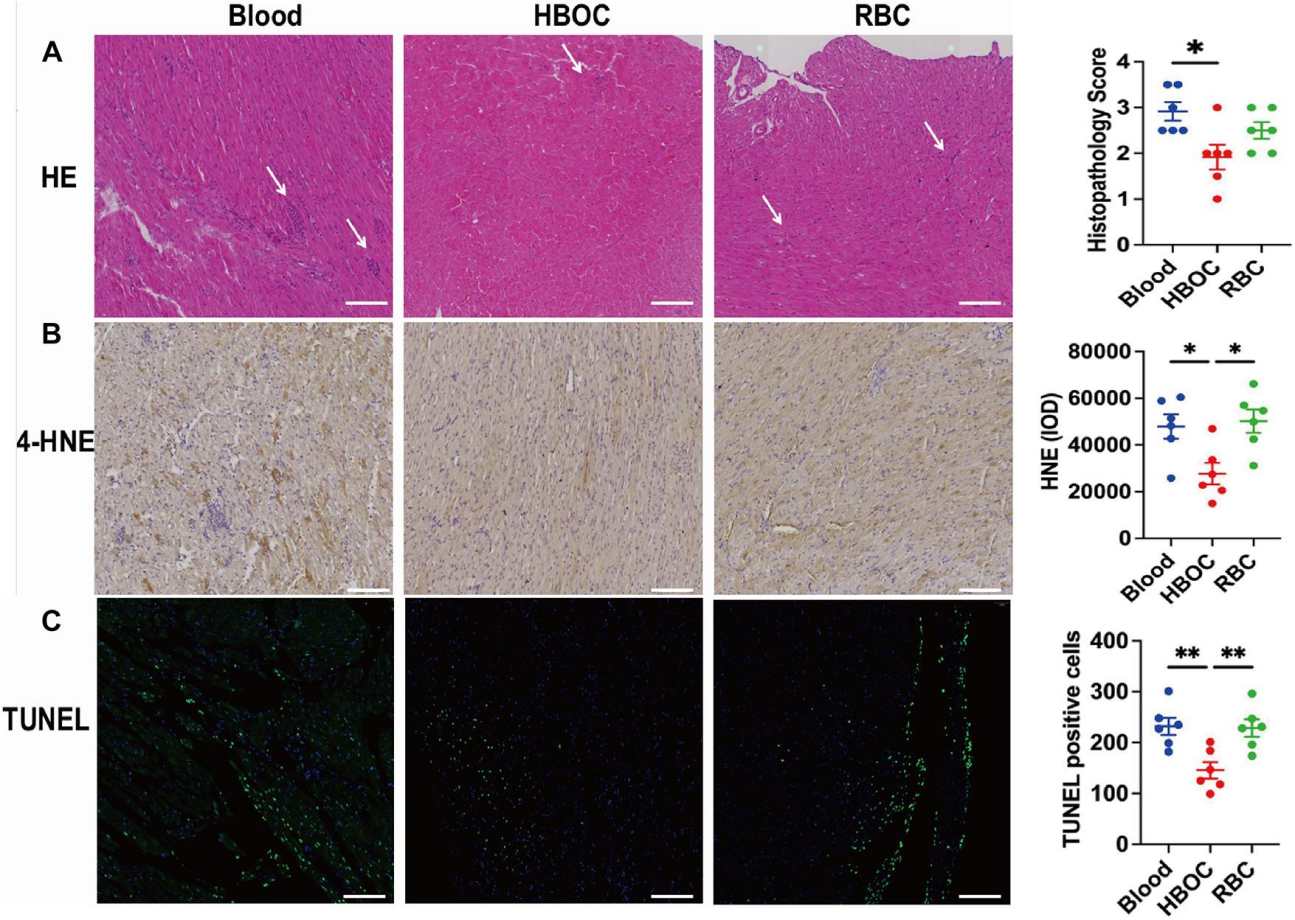
Histological analysis post-transplantation. **(A)** HE staining and injury scores; **(B)** TUNEL staining and quantification of TUNEL-positive cells; **(C)** 4-HNE immunohistochemistry. Scale ba*r* = 100 μm. *n* = 6/group. *p < 0.05; **p < 0.01.

Histological analysis performed 4 h after transplantation revealed myocardial fiber disarray, blurred cellular outlines, interstitial edema, and inflammatory cell infiltration in experimental groups (Figure 7A). The extent of inflammatory infiltration, together with myocyte necrosis and interstitial edema, was incorporated into the semi-quantitative histopathological injury scoring system. Using this composite scoring approach, grafts preserved with HBOC-based EVHP exhibited significantly lower injury scores compared with those in the blood and RBC groups, indicating overall attenuated structural damage and reduced inflammatory involvement.

Although inflammatory infiltrates were present across all groups, qualitative assessment revealed a visibly lower density and extent of infiltrating inflammatory cells in the HBOC group, whereas more prominent interstitial inflammatory accumulation was observed in the blood and RBC groups (Figure 7A).

**Figure 8:**
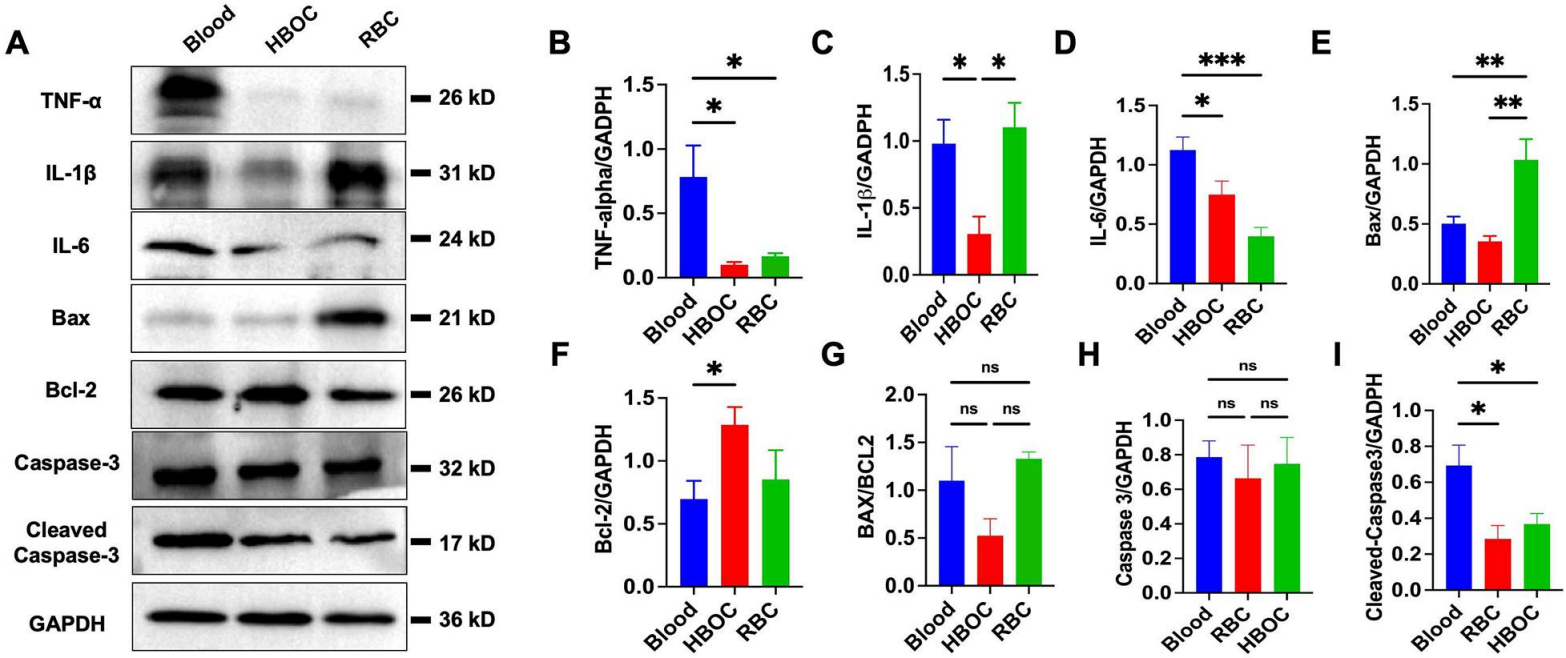
Western blot analysis post-transplantation. **(A)** Representative blots of TNF-α, IL-1β, IL-6, Bax, Bcl-2, caspase-3, and cleaved-caspase-3. **(B–I)** quantification of the protein expression. *n* = 6/group. *p < 0.05; **p < 0.01; ***p < 0.001.

Consistent with these histological findings, immunohistochemical staining showed markedly higher expression of 4-hydroxynonenal (4-HNE) in the blood and RBC groups, indicating increased oxidative stress within the graft myocardium (Figure 7B). Furthermore, western blot analysis demonstrated significantly reduced myocardial expression of the pro-inflammatory cytokines TNF-α, IL-1β, and IL-6 in the HBOC group compared with the blood and RBC groups (Figures 8A–D), supporting an attenuated inflammatory response following transplantation.

### Assessment of myocardial apoptosis post-transplantation

TUNEL staining revealed significant myocardial cell apoptosis in all three groups following perfusion. However, the number of apoptotic cells in the HBOC group was markedly lower than that observed in the blood and RBC groups (Figure 7C). Western blot analysis confirmed the expression of apoptosis-related proteins, including Bax, Bcl-2, and caspase-3, in myocardial tissues across all groups. Notably, the HBOC group exhibited significantly lower Bax expression compared to the blood and RBC groups, resulting in a reduced Bax/Bcl-2 ratio relative to the RBC group (Figures 8E,H). Furthermore, the expression level of caspase-3 in the HBOC group was significantly lower than that in the blood group (Figure 8F). To further evaluate apoptotic signaling after transplantation, cleaved-caspase 3 expression was additionally assessed in myocardial tissues harvested 4 h post-transplantation. Western blot analysis demonstrated no significant differences in cleaved-caspase 3 expression among the HBOC, blood, and RBC groups (Figure 8I), indicating comparable levels of terminal caspase activation at this post-transplant time point.

## Discussion

This study highlights the advantages of HBOC over blood- and RBC-based perfusates in DCD heart preservation. HBOC demonstrated superior oxygen delivery, maintaining higher PO_2_ levels and mitigating metabolic derangements, reflected by lower lactate and potassium concentrations. Functional analysis showed enhanced myocardial recovery in the HBOC group, with higher DP, dp/dt_max_, and dp/dt_min_. Histopathological and molecular analyses further confirmed reduced myocardial injury, inflammation, and apoptosis, as evidenced by lower levels of 4-HNE staining, decreased expression of TNF-α, IL-1β, IL-6, and a reduced Bax/Bcl-2 ratio and cleaved-caspase3 levels. These findings suggest that HBOC improves DCD heart preservation by enhancing oxygen delivery, attenuating inflammation, and minimizing oxidative and apoptotic injury.

Rebeating time differed among groups, with whole blood-perfused hearts exhibiting earlier rebeating than those in the HBOC and RBC groups. A plausible explanation is that whole blood contains plasma components, such as electrolytes and endogenous catecholamines, which may transiently facilitate early electrical and mechanical recovery after reperfusion, whereas washed RBCs and acellular HBOC-based perfusates lack these factors. Despite a relatively delayed rebeating time, HBOC-based perfusion provided more stable oxygen delivery and metabolic control during prolonged EVHP, resulting in improved downstream functional and injury-related outcomes. These proposed mechanisms remain speculative and warrant further investigation.

Originally developed as blood substitutes, HBOCs have shown broad potential in organ resuscitation and preservation (12, 13). Previous studies have reported their efficacy in *ex vivo* perfusion and IRI models. For instance, Li et al. demonstrated that polymerized porcine hemoglobin enhanced oxygenation and metabolic activity in normothermic liver perfusion (14). Similarly, Mahboub et al. reported improved kidney graft function during rewarming using HBOC-201 (12). Beyond organ perfusion, previous studies have shown protective effects of HBOCs in mitigating organ ischemia-reperfusion injury. Zhao et al. demonstrated that HBOCs reduced pulmonary vascular leakage following hemorrhagic shock (19), while Yadav et al. found that nano vascular liposome-encapsulated hemoglobin prevented multi-organ injuries in hemorrhagic shock models (20). These findings underscore the versatility of HBOCs in safeguarding organ function during critical conditions.

Our findings align with this body of research and provide additional insights into the application of HBOCs in cardiac transplantation. HBOC-based perfusate supported cardiac function recovery and mitigated oxidative stress and inflammation during *ex vivo* perfusion. Notably, HBOC-perfused hearts exhibited lower levels of inflammatory and apoptotic markers compared to blood- and RBC-based perfusates, indicating its cardioprotective role in DCD heart preservation. Importantly, transplanted hearts perfused with HBOCs demonstrated superior post-transplant function, affirming their utility in reducing myocardial ischemia-reperfusion injury. The observed benefits of HBOC can be attributed to its unique mechanisms. Firstly, HBOC efficiently carries oxygen in the absence of red blood cells, ensuring continued myocardial oxygenation during *ex vivo* perfusion. Additionally, HBOC's non-cellular nature attenuates immune activation, reducing inflammation compared to blood perfusion. This likely explains the observed reduction in inflammatory markers and tissue injury.

Our study extends the findings of White et al., who demonstrated the utilization of HBOC in porcine heart perfusion (21). In addition to assessing cardiac function and protein expression during perfusion, we transplanted the perfused hearts into recipient rats to evaluate their functional and molecular changes post-transplantation. This additional step provides a more comprehensive understanding of the viability and performance of hearts preserved with HBOC. However, discrepancies were observed between the findings of the two studies regarding cardiac function and protein expression during perfusion. A likely explanation is the difference in perfusion systems. Our study used a peristaltic pump with silicone tubing, which caused greater mechanical damage to cells in the perfusate. This damage may have contributed to the sustained elevation of potassium and lactate levels in the blood and RBC groups, compromising cardiac function. Furthermore, our study revealed unique insights into the inflammatory response. During perfusion, the blood group exhibited significant mononuclear cell infiltration into the myocardium, leading to elevated levels of inflammation-related proteins compared to the HBOC and RBC groups. This heightened inflammatory response persisted post-transplantation, resulting in more severe inflammation in donor hearts. These findings underscore the potential advantages of HBOC in reducing both acute and post-transplant inflammatory injury, positioning it as a superior alternative to traditional blood-based perfusates.

HBOCs are prone to oxidation into methemoglobin, which impairs their oxygen-carrying capacity and may exert direct cytotoxic effects on myocardial tissue (22). Our findings are consistent with previous research highlighting this issue. To mitigate methemoglobin formation, we incorporated methylene blue (MB) and glutathione (GSH) as reducing agents into the HBOC perfusate. This strategy, following the approach outlined in Zvonimir Vrselja's study on porcine brain perfusion, has been shown to effectively preserve tissue viability by controlling methemoglobin production (23). Importantly, MB and GSH likely serve a dual role in this setting. First, they stabilize HBOC by limiting methemoglobin formation, thereby preserving effective oxygen delivery during EVHP. Second, both agents possess redox-modulating and antioxidant properties that may attenuate the oxidative burst associated with early reperfusion of ischemic DCD myocardium. By simultaneously maintaining HBOC functionality and mitigating oxidative injury during the critical early reperfusion phase, the inclusion of MB and GSH likely contributed to the improved functional recovery and reduced myocardial injury observed in the HBOC-based perfusion group. These effects highlight the importance of HBOC stabilization and redox control as integral components of HBOC-based EVHP strategies, rather than ancillary additives.

### Limitations of the study and future directions

This study has several limitations. First, the perfusion system employed a peristaltic pump and silicone tubing, which may have introduced mechanical shear stress, contributing to cell damage and potentially influencing perfusion outcomes. Future optimization of EVHP platforms using alternative pump systems and less gas-permeable tubing materials may help minimize mechanical trauma and improve perfusion stability. Second, although methylene blue and glutathione effectively reduced methemoglobin formation and oxidative stress in HBOC-base perfusate, the present study was not designed to distinguish the independent contribution of HBOC-mediated oxygen delivery from the antioxidant and redox-modulating effects of these agents. Third, this study did not include a DCD control group without EVHP or a non-DCD (sham) heart control group. Preliminary experiments indicated that DCD hearts subjected to static cold preservation or cold HTK flush without subsequent EVHP exhibited severe myocardial injury and were not suitable for meaningful functional or transplantation assessment. While inclusion of a non-DCD sham control would be valuable for benchmarking the extent of EVHP-mediated recovery, the primary focus of the present study was to compare different oxygen carrier strategies within an EVHP platform specifically for DCD hearts. Future studies incorporating no-EVHP and non-DCD control groups will be important to further contextualize the protective effects observed in the HBOC-based perfusion strategy. Finally, our study limited to short-term graft function and injury. Long-term studies are needed to determine the effects of HBOC perfusion on graft survival, immunologic responses, and recipient recovery, which are critical for evaluating its clinical potential in heart transplantation.

## Conclusion

In conclusion, our study demonstrates that an HBOC-based EVHP perfusate formulated with methylene blue and glutathione improves functional recovery and attenuates myocardial injury in DCD hearts compared with perfusion using whole blood or washed red blood cells. The HBOC-based formulation was associated with enhanced graft functional performance, reduced inflammatory responses, and decreased cellular injury during *ex vivo* perfusion and following transplantation. These findings indicate that a properly stabilized HBOC-based perfusate represents a promising strategy for DCD donor heart preservation and provides important insights for the further development and optimization of EVHP protocols in translational and clinical settings.

## Data Availability

The original contributions presented in the study are included in the article/Supplementary Material, further inquiries can be directed to the corresponding author.
